# Whole-Organ Arthroscopic Knee Score (WOAKS)

**DOI:** 10.1186/1471-2474-9-155

**Published:** 2008-11-24

**Authors:** Gunter Spahn, Thomas Mückley, Hans M Klinger, Gunther O Hofmann

**Affiliations:** 1 Center of Trauma and Orthopedic Surgery Eisenach, Sophienstrasse 16, D-99817 Eisenach, Germany; 2 Department of Trauma, Friedrich Schiller University of Jena, Erlanger Allee 101, D-07740 Jena, Germany; 3 Trauma Center "Bergmannstrost" Halle, Merseburger Straße 165, D-06112 Halle, Germany; 4 Orthopaedic Center, University of Goettingen Postbox 3742, D-37070 Göttingen, Germany

## Abstract

**Background:**

To describe a semi-quantitative score for multi-feature, whole-organ evaluation of the knee in osteoarthritis based on the results of arthroscopic evaluation.

**Methods:**

This was a study of 1,199 patients who were suffering from knee pain for over 3 months (range 3 to 48 months) and had undergone arthroscopy. The mean age of patients was 49.8 (range 17 to 85) years old. Cartilage lesions were graded according to the ICRS protocol (grade 0 to 4 and for osteophytes "grade 5"). Meniscus lesions were classified regarding to the extent of resection which was needed (grade 0: intact meniscus, grade 1: partial meniscectomy, grade 2: subtotal meniscectomy, and grade 3: total meniscectomy). The whole grade of cartilage lesions was calculated as the sum of ICRS grades in all joint surfaces (bearing and non-bearing margin). The whole grade of meniscus lesions was calculated as the sum of the points for medial and lateral meniscus surgery. The Whole-Organ Arthroscopic Knee Score (WOAKS) was the sum of the cartilage and meniscus score.

**Results:**

The mean knee osteoarthritis outcome score (KOOS) of all patients was 67.3 ± 26.0 (range 21 to 128) points. The WOAKS was significantly associated (p = 0.001) with patient age (R = 0.399), the subjective complaints (R = 0.630) in KOOS, and the radiological grade of OA (R = 0.731).

**Conclusion:**

The good correlation between the WOAKS and the subjective complaints as well as the radiological grade of OA suggests that the score can be used as an instrument for description of the "whole organ" knee. This score may be useful for clinical or epidemiological studies in the future.

## Background

Arthroscopy in degenerative knee pathologies is one of the most often-performed operations in orthopedic surgery worldwide. Patients' complaints in joint degeneration respective osteoarthritis (OA) result from intraarticular lesions as well as pathologic conditions surrounding the knee (subchondral bone, mechanical axis, muscular condition etc.). Intraarticular pathologies which cause complaints and loss of function are cartilage meniscus tears in the first line.

For the grading of cartilage lesions, innumerous classifications exists [1]. General cartilage lesions are classified into four stages. At present, the ICRS classification for cartilage lesions is very popular[2].

Cartilage lesions are most often associated with meniscus lesions and vice versa.

Also, meniscus tears are a tool of the process of knee degeneration. Meniscal pathologies are often the main symptom and most often require arthroscopic interventions. The arthroscopic classification divides degenerative meniscal tears into radial and horizontal tears, flap tears, buckle-handle tears, and complex tears. It was demonstrated that the extension of meniscectomy determinatively influences the patient's complaints and the outcome. Therefore, is it possible to classify degeneration according to the need for meniscectomy (partial, subtotal, or total).

The patient's evaluation and outcome assessments after cartilage treatment can be made by using scoring systems. Innumerous scoring systems for knee evaluation are available[3,3,4]. Usual knee rating systems for outcome measurements are, for example, the IKDC (International Knee Documentation Committee form)[5,6], the Lysholm Score [7], the Tegner activity score, [8] and the Cincinnati Knee Ligament rating scale[9]. These scores were created primarily for measurements after knee injuries in younger athletic patients. For assessment in OA, these scores are less useful. Better scores in OA are based on questions about subjective complaints, activities of daily life, and lifestyle. These scores better reflect the clinical picture of OA. Well-established are the short-form health survey, which contains only 36 questions (SF-36) [10,11], the WOMAC (Western Ontario and McMaster Universities Osteoarthritis Index), [12,13] and the KOOS[14].

The visualization of degenerative joint pathologies is possible by radiography [15] and MRI [16,17].

Radiography is sufficient for the staging of osteoarthritis [18]. However, radiographic signs of degeneration (joint space narrowing, subchondral sclerosis, and osteophytes) are usually associated with a higher grade of osteoarthritis. MRI is a non-invasive method for evaluation of chondral lesions as well as meniscal tears. The MRI classification according to Peterfy et al. [19] is able to describe the knee as a "whole organ".

Until now, an analogous score ("whole organ score") for the description of the results after arthroscopic evaluation in patients with degenerative pathologies in OA does not exist.

This intent of this study was to create a score which is sufficient for the grading of joint degeneration based on arthroscopic findings in patients who were suffering from chronic degenerative knee lesions. Evaluation should be made of a possible association of such a score with the patients' subjective complaints and the radiological grade of osteoarthritis.

## Methods

### Patients

This was a study of 1,199 patients (642 male and 557 female) who were suffering from knee pain for over 3 months (11.3 ± 11.0 months, range 3 to 48 months) and who had undergone arthroscopy (Table 1). Criteria for exclusion were prior surgery, injury, osteochondritis dissecans, and rheumatic diseases. The mean patient age was 49.8 ± 15.6 (range 17 to 85) years old. All patients had given their consent written to the study. Ethical approval to perform this investigation was granted by the Jena Ethics Committee (2362-08/08).

**Table 1:** Patients

Age	n	Sex		History [month]
		Male	Female	
< 20	23	13	10	7.6 ± 7.3

20 to 29	131	80	51	7.8 ± 7.4
30 to 39	148	101	47	7.3 ± 7.1
40 to 49	277	149	128	8.5 ± 8.7
50 to 59	278	133	145	7.8 ± 4.5
60 to 69	210	93	117	18.7 ± 15.3
> 70	132	73	59	21.6 ± 11.6

In younger patients (< 40 years) significantly male patients had undergone arthroscopy, whereas in patients 50 years old and older more female patients required surgery (p < 0.001). Older patients (>60 years) had a significantly longer history (p > 0.001)

### Preoperative evaluation

The patients' complaints were elicited by using the KOOS (Knee injury osteoarthritis outcome score) in the German version in the morning of the day on which the operation was performed[20]. The KOOS questionnaire consists of 42 questions over five domains, including pain, knee symptoms, function in daily living, and function in sport and recreation, and knee-related quality of life.

Actual radiographies which were not older than 3 months were judged by author number 3. The grade of osteoarthritis (OA) was determined by the criteria of Kellgren and Lawrence [15].

### Arthroscopic operation

The arthroscopies were performed or supervised by the first author in a standard technique (general anesthesia, leg holder, and tourniquet).

**Figure 1:**
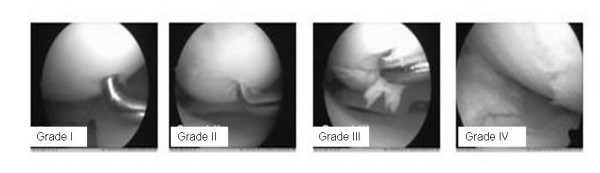
**Record of the cartilage lesions within the knee joint**. The cartilage lesions of all knees were always recorded within the same location by inspection and palpation by an arthroscopic hook. The grading was made according to the ICRS classification (range 0 to 4). Osteophytes were classified as "grade 5 lesion". Osteophytes of the trochlear grove were always found within the medial margin of the intercondylar notch (*).

**Figure 2:**
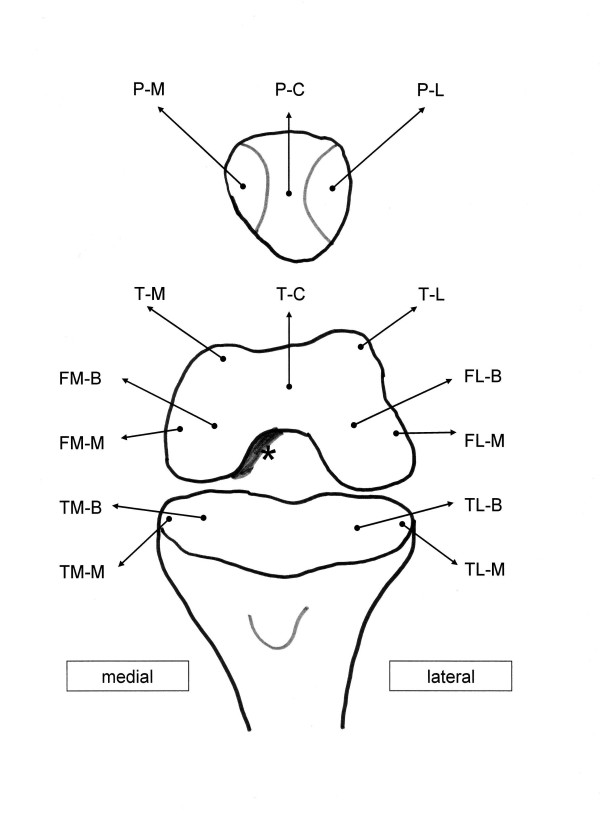
KOOS in correlation with age and gender.

The cartilage lesions were graded according to the ICRS (International Cartilage Repair Society) protocol as a result of inspection and palpation with an arthroscopic hook[2] (Figure 1). All knee compartments were inspected. The record of cartilage lesions was always made in the same locations (Figure 1). In the patella surface, the medial (P-M) and lateral margin (P-L) and the central part of the patellar crista (P-C) were judged. The trochlea was evaluated within the medial (T-M) and lateral (T-L) margin and within the trochlear groove (T-G). The chondral lesions within the medial femoro-tibial joint were evaluated within the mean bearing zone (B) and the margin (M) of the condyles and tibial surfaces: medial femoral condyle bearing zone (FM-B), medial femoral condyle margin (FM-M), medial tibial surface mean bearing zone (TM-B), and medial tibial surface margin (TM-M). Analogously, the lateral part of the femoro-tibial joint was described as FL-B, FL-M, TM-B, and TM-M (Figure 2).

**Figure 3:**
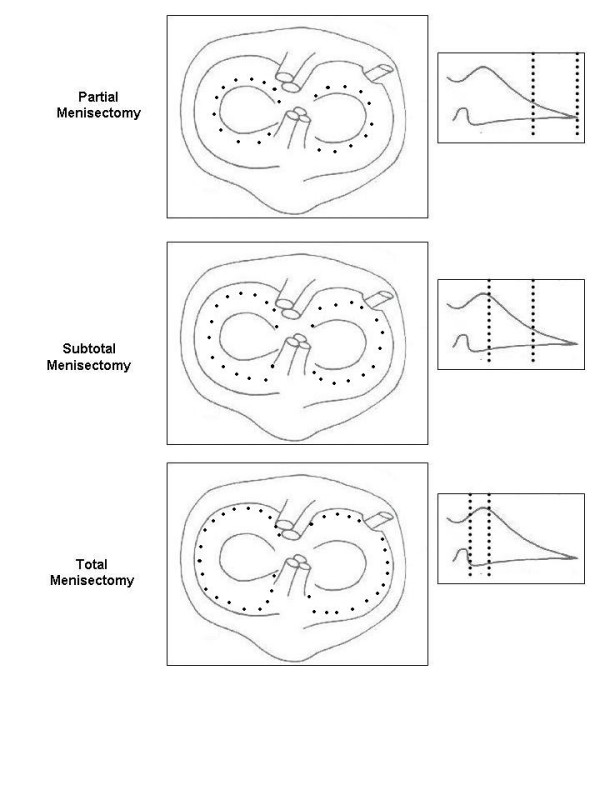
KOOS in correlation with radiological OA grade and gender.

The degrees of meniscus lesions were judged according to the required surgery: no surgery, partial meniscectomy, subtotal meniscectomy, and total meniscectomy (Figure 3).

### Arthroscopic knee score

The judged chondral surfaces were multiplicities with the ICRS grade. In case of osteophytes an additional point was added ("ICRS grade 5").

The whole grade of **cartilage lesions **was calculated as the sum of ICRS grades in all judged points: [P-M] + [P-C] + [P-L] + [T-M] + [T-G] + [T-L] + [FM-B] + [FM-L] + [TM-B] + [TM-M] + [FL-B] + [FL-M] + [TL-B] + [TL-M].

Meniscal lesions were classified accordingly to the requirement for surgical resection. The grading was made as no points for an intact meniscus, one point for a partial meniscectomy (about a third), two points for a subtotal meniscectomy (about two thirds), and three points for a total meniscectomy.

The whole grade of **meniscus lesions **was calculated as the sum of the points for medial and lateral meniscus surgery: [Medial meniscus surgery] + [Lateral meniscus surgery].

The **Whole-Organ Arthroscopic Knee Score (WOAKS) **= [Cartilage Score] + [Meniscus Score].

The score ranged from 0 points (completely intact knee) to 78 points (completely destroyed knee).

### Statistics

Statistical analyses were performed on a personal computer using the software program SPSS (13.0), SPSS Inc., Chicago Illinois. After testing for normal distribution and variance homogeneity, a One-Way Analysis of Variances (ANOVA) was preformed. Frequencies were compared by using the χ^2^-test. The Pearson correlation coefficients were used to examine the relationships between the parameters. A p value < 0.05 was considered significant.

## Results

### Knee osteoarthritis outcome score (KOOS) and radiological stage of osteoarthritis

**Figure 4:**
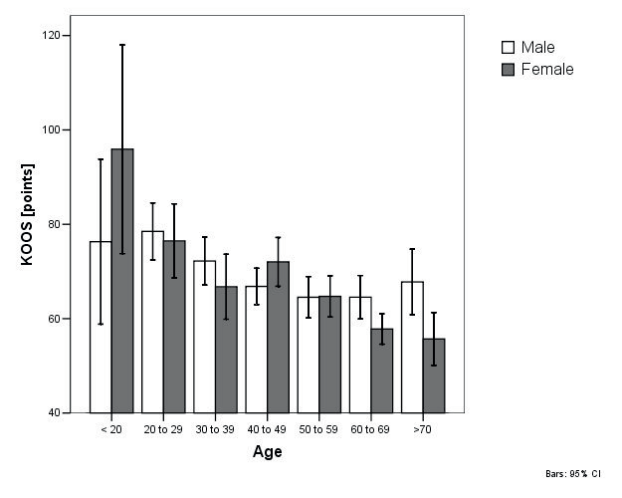
WOAKS in correlation with age and gender.

The mean KOOS of all patients was 67.3 ± 26.0 (range 21 to 128) points. The KOOS in older patients was significantly lower than in younger patients (p = 0.000), but no significant differences (p = 0.061) were found between male and female patients (figure 4).

**Figure 5:**
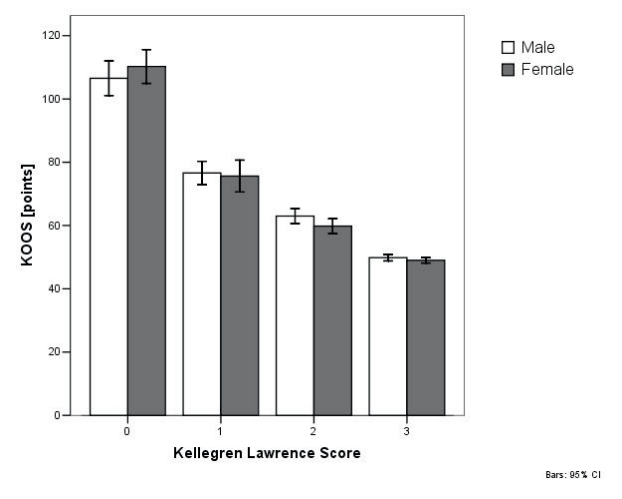
WOAKS in correlation with radiological grade of OA grade and gender.

In 171 patients (14.3%) the radiographs were without pathologies (Kellgren-Lawrence Score grade 0). In 19.4% (n = 233) grade 1 OA was found. In 426 patients (35.5%) grade 2 OA and in 369 patients (30.8%) grade 3 OA was determined. The radiological grade of osteoarthritis was significantly (p = 0.000) higher in older patients. The mean age of patients without osteoarthritis was 39.7 ± 14.0 (range 17 to 69) years. The patients were 47.3 ± 15.6 (range 18 to 84) years old in grade 1 OA, 49.2 ± 15.5 (range 20 to 84) years old in grade 2, and 56.6 ± 13.1 (range 21 to 85) years old in grade 3. Female patients tended to have a higher grade of OA (p = 0.281). There was a significant decrease (p = 0.000) in KOOS in patients with high grade OA (Figure 5).

### Arthroscopic findings

The frequencies of **cartilage lesions **within the knees are listed in Tables 2 to 5. Osteophytes were always associated with deep cartilage lesions within the according joint surface.

**Table 2:** Frequency of cartilage lesions and osteophytes of the patella

		Patella medial margin		Patella [crista]		Patella [lateral margin]	
		N	%	n	%	n	%
ICRS grade	0	560	46.7	598	49.9	534	44.5
	1	176	14.7	138	11.5	172	14.3
	2	294	24.5	267	22.3	244	20.4
	3	143	11.9	173	14.4	178	14.8
	4	17	1.4	23	1.9	68	5.7
Osteophytes	5	9	0.8			3	0.3

**Table 3:** Frequency of cartilage lesions and osteophytes of the trochlea

		Trochlea groove		Trochlea medial margin		Trochlea lateral margin	
		N	%	n	%	n	%
ICRS grade	0	600	50.0	530	44.2	524	43.7
	1	154	12.8	173	14.4	170	14.2
	2	257	21.4	247	20.6	243	20.3
	3	157	13.1	180	15.0	193	16.1
	4	19	1.6	69	5.8	69	5.8
Osteophytes	5	12	1.0				

**Table 4:** Frequency of cartilage lesions and osteophytes within the medial knee compartment

		MFC bearing zone		MFC margin		MT bearing zone		MT margin	
		N	%	n	%	n	%	n	%
ICRS grade	0	46	3,8	272	22,7	507	42,3	789	65,8
	1	119	9,9	133	11,1	44	3,7	93	7,8
	2	396	33,0	574	47,9	332	27,7	167	13,9
	3	429	35,8	200	16,7	169	14,1	135	11,3
	4	209	17,4	7	0,6	147	12,3	6	0,5
Osteophytes	5			13	1,1			9	0,8

**Table 5:** Frequency of cartilage lesions and osteophytes within the lateral knee compartment

		LFC bearing zone		LFC margin		LT bearing zone		LT margin	
		N	%	n	%	n	%	n	%
ICRS grade	0	860	71,7	921	76,8	872	72,7	931	77,6
	1	67	5,6	184	15,3	61	5,1	159	13,3
	2	183	15,3	76	6,3	159	13,3	87	7,3
	3	75	6,3	15	1,3	89	7,4	18	1,5
	4	14	1,2			18	1,5		
Osteophytes	5			3	0.2			4	0.3

Osteophytes within the lateral compartment always were associated with complete (grade 4) cartilage lesions.

The medial meniscus was intact in 251 cases. In a total of 948 cases, meniscectomy (687 times partial, 200 times subtotal, and 61 times total resection) was required. The lateral meniscus was intact in 975 knees. Surgery was needed in 224 knees: partial meniscectomy (n = 142), subtotal meniscectomy (n = 42), and total meniscectomy (n = 40). The extent of meniscus destruction correlated significantly (p = 0.001) with the radiological grade of OA (Table 6).

**Table 6:** Required meniscectomy in correlation with the grade of osteoarthritis

		**Kellegren Lawrence Score**							
		0		1		2		3	
		n	%	n	%	n	%	n	%
Medial Meniscectomy		109	63.7	52	22.3	72	16.9	18	4.9
	Partial	60	35.1	172	73.8	291	68.3	164	44.4
	Subtotal	1	0.6	8	3.4	58	13.6	133	36.0
	Total	1	0.6	1	0.4	5	1.2	36.0	14.6
Lateral Meniscectomy	Intact	169	98.8	219	94.0	376	88.3	211	57.2
	Partial	2	1.2	13	5.6	39	9.2	88	23.8
	Subtotal	0	0	1	0.4	4	0.9	37	10.0
	Total	0	0	0	0	7	1.6	33	8.9

The increase in cartilage lesions was associated with a significant (p < 0.001) decrease in KOOS (Tables 5 to 8) in all compartments.

Patients with an intact medial meniscus had a KOOS of 92.9 ± 33.5 points. In cases with a need for partial meniscectomy, the KOOS was significantly lower (64.9 ± 19.3). More excessive meniscectomy was associated with a smaller KOOS of 49.8 ± 6.1 in subtotal meniscectomy and 46.0 ± 12.5 points in total meniscectomy (p < 0.0001). Patients who needed lateral meniscectomy had a significantly (p < 0.001) smaller KOOS in partial meniscectomy (55.3 ± 17.0), subtotal meniscectomy (48.0 ± 6.3), and total meniscectomy (39.5 ± 7.1).

The presence of a joint effusion (n = 168) was associated with a higher KOOS (70 ± 19.3) than in the absence (66.7 ± 26.9) in tendency (p = 0.060). Patients with a crystal synovitis (n = 10) tended to have a lower KOOS (56.5 ± 16.0) than patients with the absence of crystals (67.4 ± 26.1), p = 0.188. Other pathologies like loose bodies (n = 58, p = 0.121), synovitis (n = 133, p = 0.559), and hypertrophic plica (n = 35, p = 0.674) did not correlate with the KOOS.

### Arthroscopic knee score

**Figure 6:**
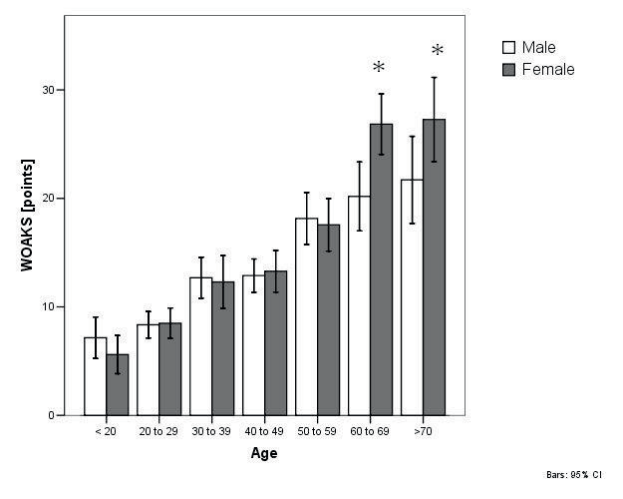
Figure 6

The mean WOAKS of all patients was 16.6 ± 13.7 (range 3 to 64 points). There was a significant correlation between WOAKS and KOOS (R = -0.630, p = 0.000) (Tables 1 to 10. The WOAKS also correlated significantly with the patient age (R = 0.399, p = 0.000). In patients who were younger than 60 years, no difference between male and female gender was found (p = 0.480). The WOAKS in female patients who were 60 years and older was significantly higher (p = 0.000, Figure 6).

**Table 7:** Correlation of cartilage lesions and osteophytes with KOOS in the patella

		Patella medial margin	Patella [crista]	Patella [lateral margin]
ICRS grade	0	84.2 ± 27.6	83.4 ± 27.5	87.0 ± 26.8
	1	57.1 ± 14.6	57.3 ± 14.5	56.3 ± 11.6
	2	51.2 ± 7.3	50.7 ± 5.3	51.1 ± 6.8
	3	50.7 ± 14.0	48.0 ± 6.7	50.1 ± 8.4
	4	47.6 ± 5.2	46.3 ± 5.2	49.0 ± 2.6
Osteophytes	5	44.8 ± 4.8		44.9 ± 7.0

**Table 8:** Correlation of cartilage lesions and osteophytes with KOOS in the trochlea

		Trochlea groove	Trochlea medial margin	Trochlea lateral margin
ICRS grade	0	83.9 ± 27.3	86,9 ± 26,9	86,6 ± 26,8
	1	53.6 ± 8.4	56,5 ± 11,6	56,3 ± 11,6
	2	50.7 ± 7.2	51,6 ± 8,6	51,1 ± 6,8
	3	48,6 ± 7,0	50,1 ± 8,3	53,1 ± 15,4
	4	49,4 ± 10,2	45,0 ± 6,9	45,0 ± 6,9
Osteophytes	5	43,7 ± 5,2		

**Table 9:** Correlation of cartilage lesions and osteophytes with KOOS in the medial compartment

		MFC bearing zone	MFC margin	MT bearing zone	MT margin
ICRS grade	0	74.1 ± 28.2	90.4 ± 34.3	88,2 ± 26.7	76.3 ± 27.7
	1	61.6 ± 17.3	60.3 ± 21.0	62.7 ± 24.1	54.2 ± 10.2
	2	74.1 ± 21.7	65.1 ± 18.4	53.7 ± 8.5	49.8 ± 4.6
	3	71.2 ± 32.0	50.3 ± 4.2	49.8 ± 4.7	49.8 ± 6.8
	4	48.3 ± 7.0	48.1 ± 8.4	47.6 ± 7.5	47.6 ± 7.5
Osteophytes	5		48.6 ± 7.4		47.0 ± 10.5

**Table 10:** Correlation of cartilage lesions and osteophytes with KOOS in the lateral compartment

		LFC bearing zone	LFC Rand	LT bearing zone	LT margin
ICRS grade	0	74.3 ± 27.5	72.8 ± 27.2	73.8 ± 27.5	72.4 ± 27.2
	1	51.7 ± 6.0	50.9 ± 6.2	52.5 ± 7.6	52.0 ± 6.7
	2	50.7 ± 6.1	49.7 ± 8.1	51.9 ± 6.7	46.4 ± 6.8
	3	45.1 ± 7.4	49.9 ± 4.5	46.2 ± 6.7	44.8 ± 4.7
	4	49.9 ± 5.5		43.3 ± 5.7	
Osteophytes	5		45.2 ± 7.5		40.5 ± 9.3

**Figure 7:**
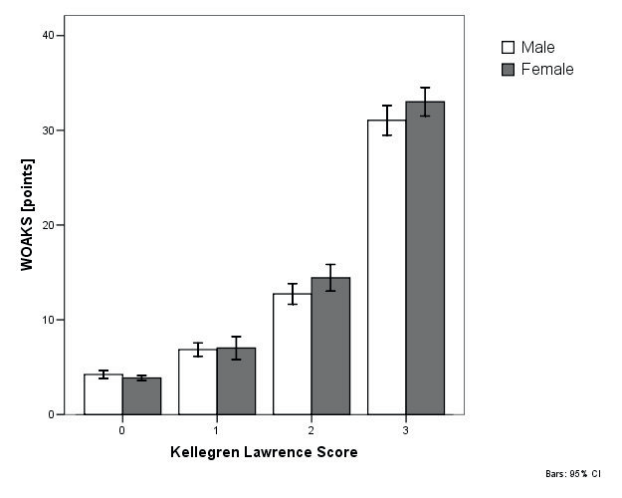
Figure 7

The WOAKS increased significantly (p = 0.000) in relation to OA grade (Figure 7). Patients with a normal radiological finding had a WOAKS of 4.1 ± 1.6 (range 3 to 11) points. In an OA grade 1, a WOAKS of 6.9 ± 4.8 (range 3 to 46) points was determined. In grade 2 OA, a WOAKS of 13.5 ± 9.3 (range 3 to 51) points was evaluated, and in grade 3 OA, a score of 32.1 ± 10.6 (range 5 to 64) was evaluated.

## Discussion

Degenerative joint disease or osteoarthritis of the knee (OA) is characterized by complex processes of degeneration within the hyaline cartilage and within the menisci. A new arthroscopic score (WOAKS) was created to estimate the degree of degeneration in the knee. There was a significant association of the WOAKS with the patients' subjective complaints as well as with the radiological stage of OA. Thus, it is sufficient for grading of degenerative joint disease respectively OA based on arthroscopic findings.

Cartilage lesions occur with four stages in arthroscopies in a high frequency[21-23]. Intact cartilage is normally smooth and has high elasticity[2]. Low grade degeneration is marked by superficial softening and fissuring (grade 1 lesions). Cartilage degeneration is associated with an increasing loss of mechanical resistance which results an increasing breakdown of the chondral layers (grade 2 lesions). In deep cartilage lesions, this breakdown involves the whole cartilage layer up to the subchondral bone (grade 3 lesions). Finally, the result is a complete loss of cartilage with widely open laying subchondral bone (cartilage defect, grade 4). The degeneration takes place primarily in the mean bearing zones of the joint. Secondary marginal cartilage zones can be involved in this degenerative process. Complete lesions of the whole joint surface which include non-weigh-bearing zones are an index for a late stage of the disease.

The loss of cartilage is associated with an increased non-physiological bearing of the subchondral bone. As a result of this bearing, the subchondral bone reacts with an increasing sclerosis and formation of osteophytes [24,25]. Osteophytes can be interpreted as a frustrated "self-healing-process" to reduce the mechanical pressure within the joint surface by increase of the bearing area [26-29]. Significant loss of cartilage, subchondral sclerosis, and osteophytes occur in the late stages of the disease and are good easy to evaluate in radiography. Radiographic classifications of OA are made by the extent of joint-space narrowing, the grade of subchondral sclerosis, and the formation of osteophytes. In our series, intraarticular osteophytes always were associated with deep cartilage lesions of the whole joint surface.

In our series, we found a good correlation between the grade of cartilage degeneration and a loss of subjective function as expressed by the KOOS. This was in accordance with the results of Kocher et al., who found a significant correlation of a decrease in Lysholm Score with the number of degenerated cartilage surfaces [30]. A good correlation was always found of the increased grade of chondral degeneration and the grade of OA in standard radiographies. The occurrence of osteophytes was significantly associated with the lowest subjective well-being expressed in the KOOS. That is why we suggested classifying osteophytes as "grade 5" lesions.

Arthroscopy is the "golden standard" for evaluation of cartilage lesions [31]. It makes it possible to inspect and probe all joint compartments. The interobserver validity of this method is generally good [32-34]. However, this classification is only able to describe the grade of cartilage lesions in a concrete lesion, not for the overall joint. Thus it is not able to describe the grade of OA in the knee as a "whole organ". The present score (WOAKS) estimates all relevant chondral surfaces, including the bearing zones on the one hand and the marginal zones on the other hand. The additional estimation of osteophytes complemented the possibility to make a diagnosis of the complete "cartilage grade".

Additional important structures which are involved in the pathway of joint degeneration are the menisci[35]. An intact meniscus reduces the pressure and shear forces within the joint surfaces, compensates for the incongruence between condyles and tibia, and promotes synovial lubrication[36].

The role of meniscus pathology in joint degeneration has been underestimated in the past. Like cartilage, the meniscal tissue undergoes advanced degenerative processes during OA development. Initially, intra-meniscal lesions (cysts, incomplete tears, and softening) are well-traceable in MRI. These initial lesions can already be associated with non-specific complaints caused by subluxation of the free margin into the joint space. The loss of mechanical resistance causes complete tears. According to Trillat's postulate, [37] the tears mostly start as longitudinal tears and can develop into the well-known tear patterns which manifest as radial, flap, buckle-handle, horizontal, or complex tears [38].

In the present study, we found no correlation between different tear patterns and subjective complaints. However, a significant association was found between the complaints and the extent of needed resection. This correlates with the results of Spahn et al., [39] who found a significantly smaller result after subtotal or total meniscectomy compared with a partial meniscetomy. Christoforakis et al. [40] initially have demonstrated that complex tears, which mostly requires extensive resections, are significantly associated with a higher grade of joint degeneration. That is why we classify the grade of meniscus degeneration accordingly to the extent of requirements in meniscus surgery.

Principally, other pathologies like loose bodies, synovialitis, effusion, and hypertrophic synovial plica can be associated with subjective complaints. In our series, we found no significant association of these pathologies with the KOOS or radiological OA grade. Therefore, these pathologies were not estimated in the WOAKS.

To our knowledge this is the first report about an arthroscopic score which makes it possible to describe the summary of all relevant degenerative pathologies in the knee. This score is based on a study of a relative high number of patients with comparable pathologies and strong criteria for exclusion. The good correlation between the WOAKS and the subjective complaints as well as the radiological grade of OA suggests that the score can be used as an instrument for description of the "whole organ" knee.

The limitation of the WOAKS in its present form is the restriction on cartilage and meniscus lesions. It may be possible to acquire more information (for example, on varus- or Valgus dysalignment, patella tracking, ligaments, or range of motion) in the future.

Future investigations are required to gather more information about the normal values of the score according to age and gender.

Thus, it could be possible to use a modified version of this score for other investigations. Multicenter studies have to be performed to evaluate the validity of this score. However, the main advantage of this method is the simple performance of the scoring and may suggest the usefulness of this method. This score may be useful for clinical or epidemiological studies in the future.

## Conclusion

The good correlation between the WOAKS and the subjective complaints as well as the radiological grade of OA suggests that the score can be used as an instrument for description of the "whole organ" knee. This score may be useful for clinical or epidemiological studies in the future.

## Competing interests

The authors declare that they have no competing interests.

## Authors' contributions

GS: Arthroscopic operations, patient selection, acquisition of data, manuscript preparation, corresponding author

TM: Statistical analysis, manuscript check,

GOH: Manuscript check

All authors read and approved the final manuscript.

## Pre-publication history

The pre-publication history for this paper can be accessed here:



## References

[B1] Higgins LD, Cole BJ, Malek MM (2004). Patient evaluation. Articular cartilage lesions A practical guide to assessment and treatment.

[B2] Brittberg M, Winalski CS (2003). Evaluation of cartilage injuries and repair. J Bone Joint Surg Am.

[B3] Marx RG, Callaghan JJ, Rosenberg AG, Rubash HE, Simonian PT, Wickiewicz TL (2003). Knee-rating scales for clinical outcome. The adult knee.

[B4] Krämer KL, Maichl FP (1993). [Scores, Bewertungsschemata und Klassifikationen in Orthopädie und Traumatologie].

[B5] Hefti F, Muller W (1993). [Current state of evaluation of knee ligament lesions. The new IKDC knee evaluation form ]. Orthopade.

[B6] Anderson AF, Irrgang JJ, Kocher MS, Mann BJ, Harrast JJ (2006). The International Knee Documentation Committee Subjective Knee Evaluation Form: normative data. Am J Sports Med.

[B7] Lysholm J, Gillquist J (1982). Evaluation of knee ligament surgery results with special emphasis on use of a scoring scale. Am J Sports Med.

[B8] Tegner Y, Lysholm J (1985). Rating systems in the evaluation of knee ligament injuries 1. Clin Orthop Relat Res.

[B9] Sgaglione NA, Del PW, Fox JM, Friedman MJ (1995). Critical analysis of knee ligament rating systems. Am J Sports Med.

[B10] Kirschner S, Walther M, Bohm D, Matzer M, Heesen T, Faller H (2003). German short musculoskeletal function assessment questionnaire (SMFA-D): comparison with the SF-36 and WOMAC in a prospective evaluation in patients with primary osteoarthritis undergoing total knee arthroplasty. Rheumatol Int.

[B11] Hawker G, Melfi C, Paul J, Green R, Bombardier C (1995). Comparison of a generic (SF-36) and a disease specific (WOMAC) (Western Ontario and McMaster Universities Osteoarthritis Index) instrument in the measurement of outcomes after knee replacement surgery. J Rheumatol.

[B12] McConnell S, Kolopack P, Davis AM (2001). The Western Ontario and McMaster Universities Osteoarthritis Index (WOMAC): a review of its utility and measurement properties. Arthritis Rheum.

[B13] Roos EM, Klassbo M, Lohmander LS (1999). WOMAC osteoarthritis index. Reliability, validity, and responsiveness in patients with arthroscopically assessed osteoarthritis. Western Ontario and MacMaster Universities. Scand J Rheumatol.

[B14] Roos EM, Roos HP, Lohmander LS, Ekdahl C, Beynnon BD (1998). Knee Injury and Osteoarthritis Outcome Score (KOOS) – development of a self-administered outcome measure 3200. J Orthop Sports Phys Ther.

[B15] Kellgren JH, Lawrence JS (1957). Radiological assessment of osteo-arthrosis. Ann Rheum Dis.

[B16] Lee KY, Dunn TC, Steinbach LS, Ozhinsky E, Ries MD, Majumdar S (2004). Computer-aided quantification of focal cartilage lesions of osteoarthritic knee using MRI. Magn Reson Imaging.

[B17] Potter HG, Foo LF (2006). Magnetic resonance imaging of articular cartilage: trauma, degeneration, and repair 1. Am J Sports Med.

[B18] Wada M, Baba H, Imura S, Morita A, Kusaka Y (1998). Relationship between radiographic classification and arthroscopic findings of articular cartilage lesions in osteoarthritis of the knee. Clin Exp Rheumatol.

[B19] Peterfy CG, Guermazi A, Zaim S, Tirman PF, Miaux Y, White D (2004). Whole-Organ Magnetic Resonance Imaging Score (WORMS) of the knee in osteoarthritis 7. Osteoarthritis Cartilage.

[B20] Roos EM, Roos HP, Lohmander LS, Ekdahl C, Beynnon BD (1998). Knee Injury and Osteoarthritis Outcome Score (KOOS) – development of a self-administered outcome measure 3200. J Orthop Sports Phys Ther.

[B21] Aroen A, Loken S, Heir S, Alvik E, Ekeland A, Granlund OG (2004). Articular cartilage lesions in 993 consecutive knee arthroscopies. Am J Sports Med.

[B22] Ritter MA, Faris PM, Thong AE, Davis KE, Meding JB, Berend ME (2004). Intra-operative findings in varus osteoarthritis of the knee. An analysis of pre-operative alignment in potential candidates for unicompartmental arthroplasty. J Bone Joint Surg Br.

[B23] Hjelle K, Solheim E, Strand T, Muri R, Brittberg M (2002). Articular cartilage defects in 1,000 knee arthroscopies. Arthroscopy.

[B24] Meachim G, Allibone R (1984). Topographical variation in the calcified zone of upper femoral articular cartilage 2. J Anat.

[B25] Mohr W (2000). Gelenkpathologie.

[B26] Kamibayashi L, Wyss UP, Cooke TD, Zee B (1995). Changes in mean trabecular orientation in the medial condyle of the proximal tibia in osteoarthritis. Calcif Tissue Int.

[B27] Wachsmuth L, Engelke K (2004). High-resolution imaging of osteoarthritis using microcomputed tomography. Methods Mol Med.

[B28] Vilalta C, Nunez M, Segur JM, Domingo A, Carbonell JA, Macule F (2004). Knee osteoarthritis: interpretation variability of radiological signs. Clin Rheumatol.

[B29] Buckland-Wright C (2004). Subchondral bone changes in hand and knee osteoarthritis detected by radiography. Osteoarthritis Cartilage.

[B30] Kocher MS, Steadman JR, Briggs KK, Sterett WI, Hawkins RJ (2004). Reliability, validity, and responsiveness of the Lysholm knee scale for various chondral disorders of the knee. J Bone Joint Surg Am.

[B31] Oakley SP, Portek I, Szomor Z, Appleyard RC, Ghosh P, Kirkham BW (2005). Arthroscopy – a potential "gold standard" for the diagnosis of the chondropathy of early osteoarthritis. Osteoarthritis Cartilage.

[B32] Friemert B, Oberlander Y, Schwarz W, Haberle HJ, Bahren W, Gerngross H (2004). Diagnosis of chondral lesions of the knee joint: can MRI replace arthroscopy? A prospective study 18. Knee Surg Sports Traumatol Arthrosc.

[B33] van KA, de Waal Malefijt MC, Jerosch J, Castro WH, Busch M, Pape M (1998). Interobserver variance in diagnostic arthroscopy of the knee. Knee Surg Sports Traumatol Arthrosc.

[B34] Jerosch J, Castro WH, de Waal Malefijt MC, Busch M, van KA (1997). [Interobserver variation in diagnostic arthroscopy of the knee joint. "How really objective are arthroscopic findings?"]. Unfallchirurg.

[B35] Bhattacharyya T, Gale D, Dewire P, Totterman S, Gale ME, McLaughlin S (2003). The clinical importance of meniscal tears demonstrated by magnetic resonance imaging in osteoarthritis of the knee. J Bone Joint Surg Am.

[B36] Abbott AE, Levine WN, Mow VC, Callaghan JJ, Rosenberg AG, Rubash HE, Simonian PT, Wickiewicz TL (2003). Biomechanics of articular cartilage and menisci of the adult knee. The adult knee.

[B37] Trillat A, Mounier-Kuhn A (1964). [Meniscal lesions during arthrosis of the knee] 2519. Rev Chir Orthop Reparatrice Appar Mot.

[B38] Strobel M (1998). Manual of arthroscopic surgery.

[B39] Spahn G, Muckley T, Kahl E, Hofmann GO (2006). Factors affecting the outcome of arthroscopy in medial-compartment osteoarthritis of the knee. Arthroscopy.

[B40] Christoforakis J, Pradhan R, Sanchez-Ballester J, Hunt N, Strachan RK (2005). Is there an association between articular cartilage changes and degenerative meniscus tears?. Arthroscopy.

